# Congenital chloride-losing diarrhea in a Mexican child with the novel homozygous SLC26A3 mutation G393W

**DOI:** 10.3389/fphys.2015.00179

**Published:** 2015-06-23

**Authors:** Fabian R. Reimold, Savithri Balasubramanian, David B. Doroquez, Boris E. Shmukler, Zsuzsanna K. Zsengeller, David Saslowsky, Jay R. Thiagarajah, Isaac E. Stillman, Wayne I. Lencer, Bai-Lin Wu, Salvador Villalpando-Carrion, Seth L. Alper

**Affiliations:** ^1^Renal Division, Beth Israel Deaconess Medical CenterBoston, MA, USA; ^2^Department of Pathology, Beth Israel Deaconess Medical CenterBoston, MA, USA; ^3^Department of Pathology, Harvard Medical SchoolBoston, MA, USA; ^4^Division of Pediatric Gastroenterology, Boston Children's HospitalBoston, MA, USA; ^5^Department of Pediatrics, Harvard Medical SchoolBoston, MA, USA; ^6^Harvard Digestive Diseases Center, Harvard Medical SchoolBoston, MA, USA; ^7^Genetics Diagnostic Laboratory and Claritas Genetics, Boston Children's HospitalBoston, MA, USA; ^8^Children's Hospital and Institute of Biomedical Sciences of Fudan UniversityShanghai, China; ^9^Department of Pediatric Gastroenterology and Nutrition, Hospital Infantil de Mexico Federico GomezMexico City, Mexico; ^10^Department of Medicine, Harvard Medical SchoolBoston, MA, USA

**Keywords:** chloride bicarbonate exchange, down-regulated in adenoma, NaCl reabsorption, Xenopus oocyte, MDCK cells

## Abstract

Congenital chloride diarrhea is an autosomal recessive disease caused by mutations in the intestinal lumenal membrane Cl^−^/HCO^−^_3_ exchanger, SLC26A3. We report here the novel SLC26A3 mutation G393W in a Mexican child, the first such report in a patient from Central America. SLC26A3 G393W expression in Xenopus oocytes exhibits a mild hypomorphic phenotype, with normal surface expression and moderately reduced anion transport function. However, expression of HA-SLC26A3 in HEK-293 cells reveals intracellular retention and greatly decreased steady-state levels of the mutant polypeptide, in contrast to peripheral membrane expression of the wildtype protein. Whereas wildtype HA-SLC26A3 is apically localized in polarized monolayers of filter-grown MDCK cells and Caco2 cells, mutant HA-SLC26A3 G393W exhibits decreased total polypeptide abundance, with reduced or absent surface expression and sparse punctate (or absent) intracellular distribution. The WT protein is similarly localized in LLC-PK1 cells, but the mutant fails to accumulate to detectable levels. We conclude that the chloride-losing diarrhea phenotype associated with homozygous expression of SLC26A3 G393W likely reflects lack of apical surface expression in enterocytes, secondary to combined abnormalities in polypeptide trafficking and stability. Future progress in development of general or target-specific folding chaperonins and correctors may hold promise for pharmacological rescue of this and similar genetic defects in membrane protein targeting.

## Introduction

Congenital chloride diarrhea (CLD; OMIM# 214700) is a rare chronic secretory diarrhea of autosomal recessive inheritance associated with polyhydramnios and premature birth. Postnatal clinical diagnosis is based on the presentation of dehydration and failure to thrive in the setting of hypokalemic metabolic alkalosis, with acidic stool pH and elevated stool chloride (>90 mM) measured after normalization of systemic volume status and serum electrolytes. Untreated disease leads to chronic systemic volume depletion, nephrocalcinosis and impaired renal function sometimes progressing to end-age renal disease. Additional clinical problems noted later in life have included intestinal inflammation, hyperuricemia, inguinal hernia, and impaired male fertility (Wedenoja et al., 2011).

CLD is caused by mutations in the gene encoding SLC26A3 (Hoglund et al., 1996), with 21 exons spanning ~38 kb on chromosome 7q31.1. The single ~2900 bp mRNA SLC26A3 transcript encodes a polypeptide of 764 amino acids (aa) in length. The SLC26A3 polypeptide includes a short N-terminal cytoplasmic domain, a ~500 aa polytopic transmembrane domain of unknown structure that has been tentatively modeled on the transmembrane domain of the ecCLC Cl^−^/H^+^ antiporter (Ohana et al., 2011), and a ~220 aa C-terminal cytoplasmic region comprising mainly the Sulfate Transporter and Anti-Sigma Factor Antagonist (STAS) domain (Sharma et al., 2011). SLC26A3 mediates Cl^−^/HCO^−^_3_ exchange across the apical membrane of enterocytes, coordinating with Na^+^/H^+^ exchanger NHE3 (and in some conditions, NHE8, Xu et al., 2012) to carry out electroneutral NaCl reabsorption from the intestinal lumen (Zachos et al., 2005; Kato and Romero, 2011).

CLD incidence is as high as 1 in 3200 births in Kuwait and other Persian Gulf countries, associated with high rates of consanguinity. Moderately elevated rates have also been noted in Finland (1 in 30–40,000 births), and Poland (1 in 200,000 births). Isolated cases have been reported in many other countries (Wedenoja et al., 2011). Here we report a novel missense mutation in the transmembrane domain of SLC26A3, in the context of the first SLC26A3-linked CLD case reported from Mexico. The mutant protein as expressed in Xenopus oocytes accumulates to normal levels and exhibits only modest loss-of-function. However, when expressed in HEK-293 cells, MDCK cells, LLC-PK1 cells, or Caco2 cells, the mutant protein accumulates to low or undetectable levels, with reduced or absent cell surface expression. We conclude that the G393W missense substitution likely causes CLD by destabilization and/or mistargeting of the SLC26A3 polypeptide.

## Methods

### Genomic DNA preparation and sequencing

Blood was obtained from the patient and his parents and subjected to genetic analysis according to protocols approved by the Committees on Clinical Investigation of the Hospital Infantil de Mexico Federico Gomez, of Beth Israel Deaconess Medical Center, and of Boston Children's Hospital. Whole blood sent from Mexico City to Boston was subjected to genomic DNA extraction using the QIAamp DNA Blood Mini Kit (Qiagen, Hilden, Germany). Oligonucleotide primers for amplification of exons and flanking regions of the candidate disease gene SLC26A3/DRA were designed with Primer3 software (SimGene.com), and are available upon request. Amplimers were subjected to Sanger sequencing on both strands.

### cRNA transcription and expression in xenopus oocytes

SLC26A3 cDNA in Xenopus oocyte expression vector pBF (Chernova et al., 2003) was subjected to missense mutagenesis to generate the G393W mutant cDNA, using the four-primer polymerase chain reaction (PCR) method (oligonucleotide primer sequences available upon request). Capped SLC26A3 cRNA was transcribed from linearized plasmid DNA template with SP6 RNA polymerase (Megascript, Ambion, Austin, TX) and purified with an RNeasy mini-kit (Qiagen, Germantown, MD). cRNA concentration (A_260_) was measured by Nanodrop spectrometer (ThermoFisher, Waltham, MA), and integrity was confirmed by formaldehyde agarose gel electrophoresis. Mature female Xenopus laevis frogs (Dept. of Systems Biology, Harvard Medical School) were subjected to partial ovariectomy under hypothermic tricaine anesthesia following protocols approved by the Institutional Animal Care and Use Committee (IACUC) of Beth Israel Deaconess Medical Center. Stage V-VI oocytes were prepared by overnight incubation of ovarian fragments in 1.5 mg/ml collagenase B (Alfa Aesar, Ward Hills, MA) dissolved in MBS (in mM: 88 NaCl, 1 KCl, 2.4 NaHCO_3_, 0.82 MgSO_4_, 0.33 Ca(NO_3_)_2_, 0.41 CaCl_2_, and 10 HEPES, pH 7.40), followed by a 20 min rinse in Ca^2+^-free MBS, with subsequent manual selection and defolliculation as needed. Oocytes were injected on the same day with cRNA (10 ng or as indicated), and maintained 72 h at 17.5°C in MBS containing 10 μg/mL gentamicin until used for experiments. As uninjected and water-injected oocytes did not differ in anion transport at 72 h post-injection (data not shown), uninjected oocytes were used as controls for the current experiments.

### ^36^Cl^−^ influx assays in xenopus oocytes

Unidirectional ^36^Cl^−^ influx studies were carried out for periods of 30 min in 148 μL ND-96 (in mM: 96 NaCl, 2 KCl, 1.8 CaCl_2_, 1 MgCl_2_, and 5 HEPES, pH 7.40) containing 2 μL carrier-free 260 mM Na^36^Cl (0.25 μCi), resulting in total bath [Cl^−^] of 103.6 mM. In Cl^−^-substituted solutions, NaCl was replaced mole-for-mole with NaHCO_3_ or Na cyclamate. Cl^−^ salts of K^+^, Ca^2+^, and Mg^2+^ were substituted on an equimolar basis with the corresponding gluconate salts as needed. All ^36^Cl^−^ influx solutions contained 10 μM bumetanide to block Cl^−^ flux via native oocyte NKCC1. Uptake was linear over 30 min under these conditions.

Oocyte ^36^Cl^−^ influx experiments were terminated with four washes in ice-cold isotonic Na cyclamate solution. Washed oocytes were individually lysed in 150 μL 2% sodium dodecyl sulfate (SDS). Triplicate 10 μL aliquots of the influx solution were used to calculate specific activity of radiolabeled substrate anions. Oocyte anion uptake was calculated from cpm values of washed oocytes and from bath specific activity.

### ^36^Cl^−^ efflux assays in xenopus oocytes

Individual oocytes were injected with 50 nl of 260 mM Na^36^Cl (6.25 nCi, 20,000–24,000 cpm). Following a 5–10 min recovery period in Cl^−^-free cyclamate solution, the efflux assay was initiated by transfer of individual oocytes to 6 ml borosilicate glass tubes, each containing 1 ml efflux solution with the indicated anions and 10 μM bumetanide. At intervals of 3 min, 0.95 ml of this efflux solution was removed for scintillation counting and replaced with an equal volume of fresh efflux solution. Following completion of the assay with a final efflux period in Cl^−^-free cyclamate solution, each oocyte was lysed in 150 μL of 2% SDS. Samples were counted for 3–5 min such that the magnitude of 2SD was <5% of the sample mean.

Efflux data was plotted as the natural logarithm (ln) of the quantity (% cpm remaining in the oocyte) vs. time. Efflux rate constants for ^36^Cl^−^ were measured from linear fits to data from the last three time points sampled within each experimental period. For each experiment, uninjected oocytes from the same frog were subjected to parallel measurements with cRNA-injected oocytes. Oocytes with <15% of injected ^36^Cl^−^ remaining at the end of the assay were excluded from analysis.

### Confocal immunofluorescence microscopy of xenopus oocytes

Oocytes were injected with 10 ng cRNA encoding wild-type or mutant SLC26A3 bearing at their N-termini the HA epitope tag (YPYDVPDYA). Uninjected oocytes and oocytes injected with cRNA 3 days prior were fixed at 4°C for 30 min in phosphate-buffered saline (PBS) containing 1.5% paraformaldehyde (PFA), then washed three times in PBS supplemented with 0.002% sodium azide, exposed to 1% SDS in 1x PBS for 5 min, and then blocked in PBS with 1% bovine serum albumin (BSA) and 0.05% saponin for 1 h at 4°C. Oocytes were then incubated 2 h at 4°C with rabbit monoclonal anti-HA peptide (dilution 1:1600; Cell Signaling, Danvers, MA), followed by three washes with 1% PBS-BSA. Oocytes were then incubated for 2 h with Cy3-conjugated secondary donkey anti-rabbit Ig (dilution 1:1600; Jackson Immunochemicals, West Grove, PA) and again thoroughly washed in PBS-BSA. Oocytes were aligned in uniform orientation along a plexiglass groove and sequentially imaged through the 10X objective of a Zeiss LSM510 laser scanning confocal microscope, using the 543-nm laser line at 512 × 512 resolution, at constant filter, gain, and pinhole settings.

Polypeptide abundance at or near each oocyte surface was estimated by quantitation of specific fluorescence intensity (FI) at the periphery of one quadrant of an equatorial focal plane (Image J v. 1.38, National Institutes of Health). The mean background FI of uninjected oocytes was subtracted from each single oocyte FI. Normalized means and standard errors were calculated for each group. Images of median intensity were selected from each group for presentation.

### SLC26A3 expression in mammalian cells

Wildtype and G393W mutant SLC26A3 cDNAs encoding an N-terminal HA epitope tag were subcloned into eukaryotic expression vector pcDNA3. All cells were grown at 37°C in humidifed 5% CO2. HEK-293T cells maintained in DMEM + 10% calf serum were grown to 80–90% confluency on 24 mm glass coverslips. MDCK cells maintained in DMEM + 10% calf serum were grown to 80–90% confluency or to full confluency on 12 mm Transwell polycarbonate inserts (Corning Life Sciences, Lowell, MA) placed in 12-well plates. Cells in each well were transfected with 1 μg cDNA using Lipofectamine 2000 (Life Technologies, Carlsbad, CA). Caco2 cells maintained in high glucose DMEM supplemented with 10% FBS and 15 mM HEPES, pH 7.4, were grown to low confluency or near-confluency on glass coverslips, or to confluency on Transwell inserts, and transfected with 1 μg cDNA using either Lipofectamine 2000 or X-Fect (Takara ClonTech). LLC-PK1 cells maintained in Medium 199 supplemented with L-glutamine, 2.2 g/L sodium bicarbonate, and 3% FBS were grown to near-confluency on glass coverslips previously coated with Synthemax (Corning Life Sciences, Tewksbury, MA). 10^6^ cells were transfected with 2 μg cDNA with the Amaxa Nucleofector II (Lonza, Allendale, NJ) using Kit L and Program T-020, as per manufacturer's protocol. After 24–72 h, PBS-rinsed cells were fixed 10 min in 2% paraformaldehyde, then washed three times with phosphate-buffered saline (PBS: 140 mM NaCl, 20 mM NaHPO_4_, pH 7.40). If not processed immediately, fixed cells were stored at 4°C until further use. Rabbit anti-HA was from Cell Signaling Technologies. Mouse anti-gp135/podocalyxin was a gift of George Ojakian. Rabbit anti-α1-Na,K-ATPase was from Santa Cruz Biotechnology. Mouse anti-α1-Na,K-ATPase was from Developmental Studies Hybridoma Bank (Iowa City, IA). Anti-E-Cadherin was from BD Transduction Laboratories (San Jose, CA). F-actin was detected with AlexaFluor647-phalloidin (Molecular Probes). Nuclear DNA was detected with DAPI or DRAQ-5 (Thermo Scientific, Life Technologies) as indicated.

### SLC26A3 immunoblot

HEK-293 cells transiently transfected as above with HA-SLC26A3 cDNA were maintained for 24 h, then lysed in RIPA buffer containing 150 mM NaCl, 50 mM Tris pH 7.4, 1% NP-40, 1% Na deoxycholic acid, and 0.1% SDS. The cell lysate was cleared by centrifugation at 4°C, and protein concentration of the cleared lysate was measured by BCA assay (Pierce Biotechnology, Rockford, IL). 50 μg total protein in 1X sample loading buffer supplemented with 5% β-mercaptoethanol denatured for 5 min at 95°C, then loaded into each lane of a precast 7.5% polyacrylamide gel (BioRad Laboratories, Hercules, CA) and fractionated by SDS-PAGE. Semi-dry transfer onto a PVDF membrane was followed by blocking the membrane for 1 h at 4°C in 1X PBS containing 5% nonfat dry milk. The membrane was incubated overnight at 4°C in with rabbit monoclonal anti-HA peptide (1:1000; Cell Signaling, Danvers, MA), washed in PBS containing 1% Tween, then incubated in horseradish peroxidase (HRP)-coupled goat-anti-rabbit Ig (1:2000; Cell Signaling). After thorough washing, the membrane was exposed for 1 min to enhanced chemiluminescence (ECL) media (GE Healthcare Life Sciences, Pittsburgh, PA), followed by 5–10 s exposure to HyBlot CL Autoradiography film (Denville Scientific, Metuchen, NJ). The film was then developed.

### Confocal immunofluorescence microscopy of mammalian cells

Fixed cells transfected with WT or mutant HA-SLC26A3 were blocked for 45 min with 7% normal horse serum, then incubated overnight at 4°C with monoclonal anti-HA (1:300, Cell Signaling Technologies), washed three times in PBS, then incubated 1 h at room temperature with Alexa 546-coupled goat anti-rabbit Ig (Life Technologies). After three final washes, coverslips were inverted and mounted on glass slides using VECTASHIELD mounting medium (Vector Labs, Burlingame, CA). Other antigens were detected with compatible, species-specific fluorophor-coupled secondary Ig. After fixation and immunostaining of filter-grown cells as described above, the filters were excised from their frames and mounted on glass slides under coverslips with VECTASHIELD. Cells were imaged with the Zeiss LSM510 confocal fluorescence microscope or with the Nikon TE2000 inverted microscope interfaced to a PerkinElmer spinning disk confocal unit and an Orca charge-coupled device camera (Hamamatsu, Bridgewater, NJ). Image processing was with Zeiss software or (for images captured by Nikon-PerkinElmer microscope) with Slidebook (Intelligent Imaging Innovations, Denver, CO).

## Results

### Clinical data

The proband was a male infant, born in the setting of polyhydramnios noted at gestational week 34. He exhibited normal appetite and weight gain during the initial postnatal weeks. At age 2 months he was hospitalized in a local Mexican hospital with acute diarrhea requiring intravenous rehydration therapy. Five additional hospitalizations for intravenous rehydration treatment of diarrhea led to referral to Mexico City's Hospital Infantil for further evaluation. The patient was found to have hypochloremic metabolic alkalosis with hypokalemia and elevated serum levels of renin and aldosterone. These abnormalities were responsive to intravenous fluid therapy and a period of total parenteral nutrition. Infectious causes of diarrhea were ruled out. Cystic fibrosis (CF) sweat tests were negative on two occasions. Complete endoscopic evaluation with biopsy revealed normal colonic mucosa by light and electron microscopy, and normal gastric and duodenal mucosae by light microscopy (data not shown). In the euvolemic state, fecal [Cl^−^] was 160 mM on two separate occasions. A clinical diagnosis of congenital chloride-losing diarrhea was made. After nearly 4 weeks of total parenteral nutrition, the patient was weaned onto food supplemented with oral KCl and NaCl, with subsequent acceleration of weight gain and growth rate.

### Molecular diagnosis

**Figure 1:**
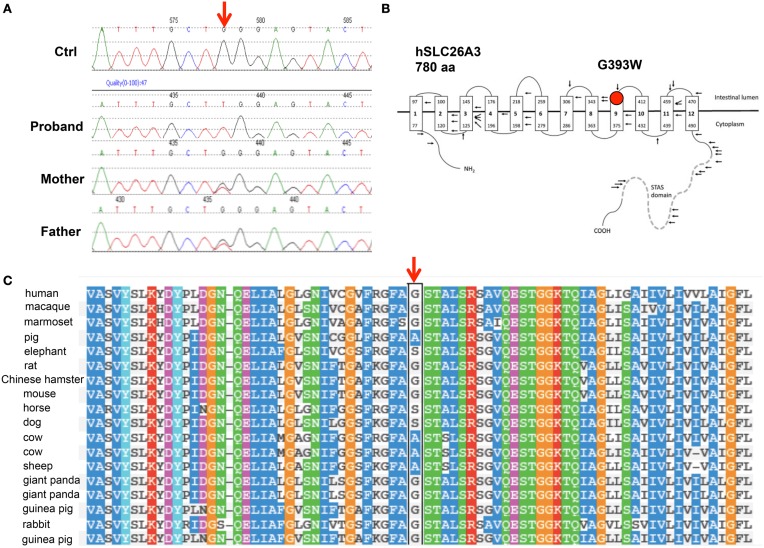
**The novel SLC26A3 mutation G393W in a child with chloride-losing diarrhea. (A)** Genomic DNA Sequence phoretograms of proband and parents. **(B)** Topographic model of hSLC26A3 (reproduced from Wedenoja et al., 2011) showing the predicted location of G393 within the transmembrane domain. **(C)** Alignment of mammalian SLC26A3 polypeptide sequences in the region of hSLC26A3 G393 (black box), showing partial conservation among species orthologs, with substitutions restricted to Ala and Ser (Polyphen-2 multiple sequence alignment).

SLC26A3 was considered a strong candidate gene for harboring a coding mutation that might explain the chronic, severe diarrhea of early onset in this child. DNA sequencing indeed revealed the novel, homozygous *SLC26A3* mutation c.1177G>T encoding the missense substitution p.Gly393Trp (G393W, Figure 1A). This mutation was absent from the 1000 Genomes Project and from the NHLBI Exome Project. The G393W mutation is predicted to be “probably damaging” by PolyPhen-2 (scores 0.95 and 0.99 with two models). Amino acid residue G393 resides within the polytopic transmembrane domain of SLC26A3 (Figure 1B). Although the membrane domains of SLC26 polypeptides are of unknown topographical disposition, hydropathy profiling has predicted a location for G393 at the exofacial end of putative transmembrane span 9 (Wedenoja et al., 2011). This residue is conserved among SLC26A3 orthologs in primates, giant panda, and rodents. However, G393 is substituted by Ala in pig, cow, and sheep, and by Ser in dog, horse, and elephant (Figure 1C). The residue is not conserved in *X. laevis* SLC26A3 or among human SLC26 paralogs (not shown).

The patient was also homozygous for the common synonymous single nucleotide polymorphism rs3735605 (c.1299G>A/p.Ala433Ala; minor allele frequency 0.159) and the rare polymorphism rs41669 (c.1953T>C/p.Leu651Leu; minor allele frequency 0.0023). Both parents were heterozygous for G393W and for the two synonymous polymorphisms.

### SLC26A3 G393W exhibits reduced anion transport function in xenopus oocytes

**Figure 2:**
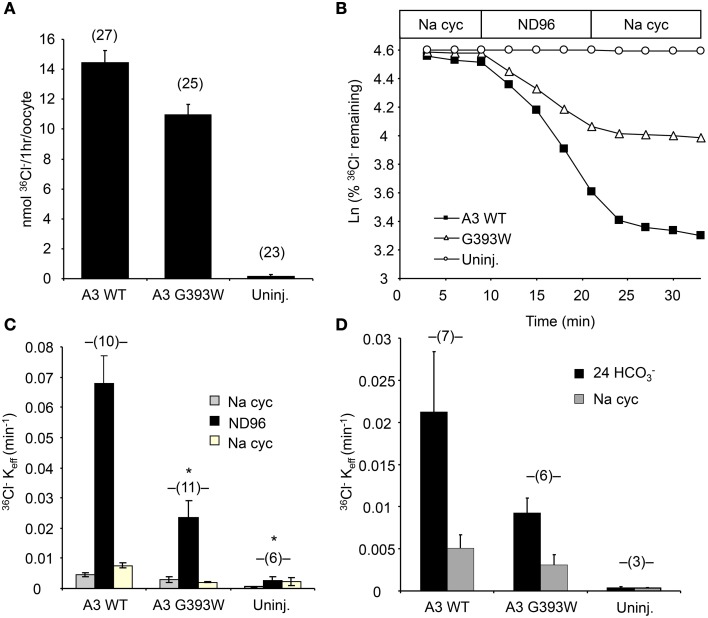
**SLC26A3 mutant G393W expressed in Xenopus oocytes exhibits modest loss-of-function. (A)** ^36^Cl^−^ influx into (n) uninjected oocytes (uninj.) or oocytes previously injected with 10 ng cRNA encoding hSLC26A3 WT (A3 WT) or its mutant hSLC26A3 G393W (A3 G393W). WT and mutant did not differ as judged by One-Way ANOVA analysis, but transport by G393W-expressing oocytes was significantly reduced as judged by Student's *T*-Test (*p* < 0.001). **(B)** ^36^Cl^−^ efflux traces of representative individual oocytes previously uninjected (Uninj, open circles) or injected with 10 ng cRNA encoding hSLC26A3 WT (A3 WT, black squares) or mutant hSLC26A3 G393W (A3 G393W, open triangles), during sequential exposures to baths of sodium cyclamate (Na cyc.), ND-96, and Na cyc. **(C)** Summarized ^36^Cl^−^ efflux rate constant data from (n) oocytes subjected to the protocol presented in **(B)**. Values are means ± s.e.m. ^*^p < 0.05 vs. hSLC26A3 WT (One-Way ANOVA). **(D)** Summarized ^36^Cl^−^ efflux rate constant data from (n) uninjected oocytes (Uninj.) or oocytes previously injected with 10 ng of cRNA encoding hSLC26A3 WT or mutant G393W exposed first to 24 mM NaHCO_3_ plus 72 mM Na cyc (Cl^−^/HCO^−^_3_ exchange), followed by 96 mM Na cyc. ^36^Cl^−^ efflux into HCO^−^_3_ solution, corrected for that into HCO^−^_3_-free cyclamate solution, did not differ statistically among groups.

**Figure 3:**
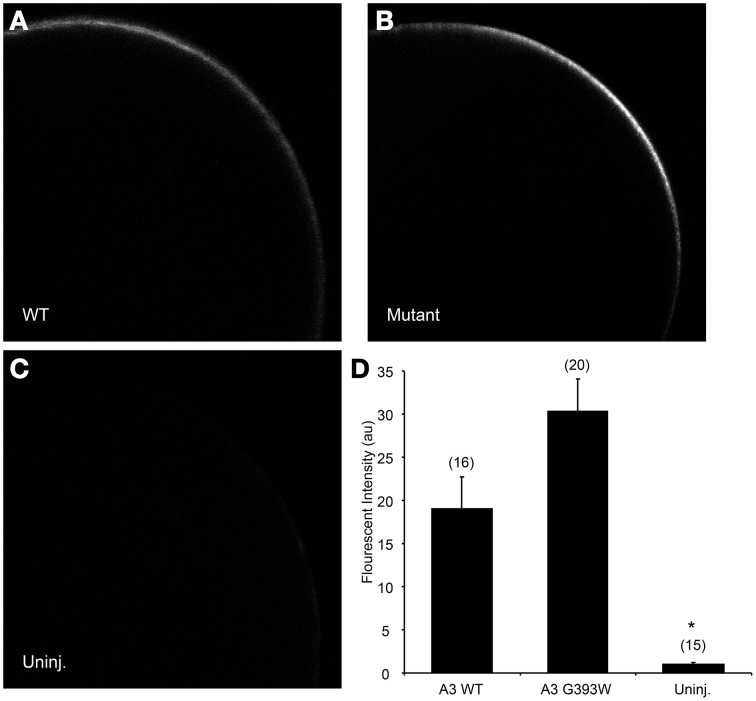
**SLC26A3 localization in Xenopus oocytes**. Median intensity examples of confocal immunofluorescence images of Xenopus oocytes previously injected with 10 ng cRNA encoding **(A)** N-terminally HA-tagged wildtype hSLC263, **(B)** HA-tagged hSLC26A3 mutant G393W, or **(C)** oocytes previously uninjected with cRNA. **(D)** Mean values (± s.e.m.) of normalized fluorescent intensity (FI) for (n) oocytes similar to those presented in **(A–C)**. FI did not differ between WT and mutant hSLC26A3-expressing oocytes, but both differed from the uninjected group (^*^p < 0.05).

cRNA encoding SLC26A3 was transcribed and injected into Xenopus oocytes. After 72 h, oocytes were subjected to measurements of unidirectional ^36^Cl^−^ influx. As shown in Figure 2A, uptake of ^36^Cl^−^ into oocytes injected with the mutant cRNA was reduced only slightly as compared to oocytes injected with wildtype SLC26A3 cRNA. The measured ^36^Cl^−^ uptake operationally represented anion exchange, since ^36^Cl^−^ efflux mediated by both wildtype and mutant SLC26A3 exhibited trans-anion dependence (Figures 2B,C). The ^36^Cl^−^ efflux activity of SLC26A3 G393W-expressing oocytes was reduced to a greater degree than was influx into similarly injected oocytes. Oocytes expressing SLC26A3 G393W mediated Cl^−^/HCO^−^_3_ exchange at reduced rates (Figure 2D) proportionate to the reduction in Cl^−^/Cl^−^ exchange (Figure 2C). The reduction in transport activity by SLC26A3 G393W was likely intrinsic to the mutant polypeptide, since Xenopus oocyte surface expression of the HA-tagged mutant polypeptide is undiminished compared to that of wildtype SLC26A3 (Figure 3).

### SL26A3 G393W is mistargeted and fails to accumulate in HEK-293 cells

**Figure 4:**
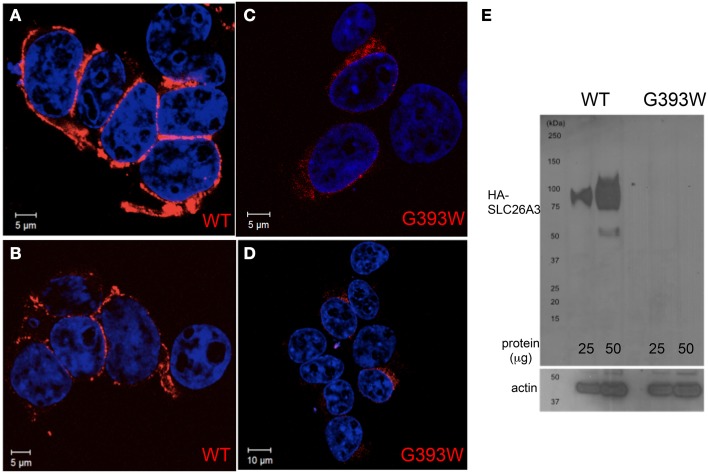
**SLC26A3 localization in HEK-293 cells**. HEK-293 cells 48 h after transient transfection with cDNA encoding HA-SLC26A3 wildtype **(A,B)** or mutant HA-SLC26A3 G393 **(C,D)**. HA epitope is red, and nuclei are stained with DAPI. **(E)** Immunoblot of whole cell RIPA lysates from HEK-293 cells prepared 24 h post-transient transfection with cDNA encoding HA-SLC26A3 wildtype (left two lanes) or mutant HA-SLC26A3 G393W (right two lanes). Actin immunoblot serves as load controls (below).

The 35–80% decrease in anion transport function exhibited in Xenopus oocytes is likely inadequate to account for the severity of the proband's clinical phenotype. We therefore transiently expressed SLC26A3 G393W in HEK-293 cells. As shown in Figures 4A,B, HA-tagged wildtype SLC26A3 localized primarily to the cell periphery, whereas the HA-tagged mutant polypeptide was detected in smaller numbers of cells, in which it accumulated in a perinuclear distribution without reaching the cell periphery (Figures 4C,D). Assessment of total SLC26A3 polypeptide accumulation by immunoblot of total cell lysates (Figure 4E) revealed that the robust accumulation of wildtype protein was not reproduced by the G393W mutant polypeptide, which was below the immunoblot detection threshold. These results suggested that the G393W polypeptide is synthesized in HEK-293 cells, but is unstable.

### SLC26A3 G393W accumulation is reduced and transient in Caco-2 cells

**Figure 5:**
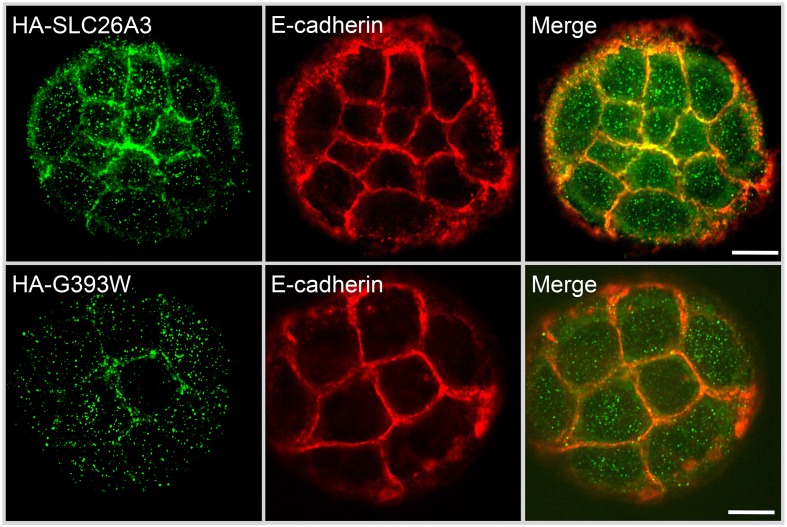
**Localization of wildtype and mutant SLC26A3 polypeptides in nonconfluent, glass-grown Caco-2 cells**. HA-SLC26A3 WT (upper panels) and HA-SLC26A3 G393G in subconfluent Caco-2 cells on glass, 24 h post-transient transfection, Cells were stained as indicated with anti-HA (left) and with anti-E-cadherin (middle). Merged images shown at right. Scale bar, 10 μm.

Figure 5 shows that in small colonies of Caco-2 cells grown on glass, at 24 h post-transfection, WT HA-SLC26A3 localizes predominantly to the peripheral membrane, where it partially colocalizes with E-cadherin, in addition to lesser accumulation in a diffuse punctate distribution. In contrast, HA-SLC26A3 G393W exhibits only a diffuse punctate distribution at reduced abundance.

**Figure 6:**
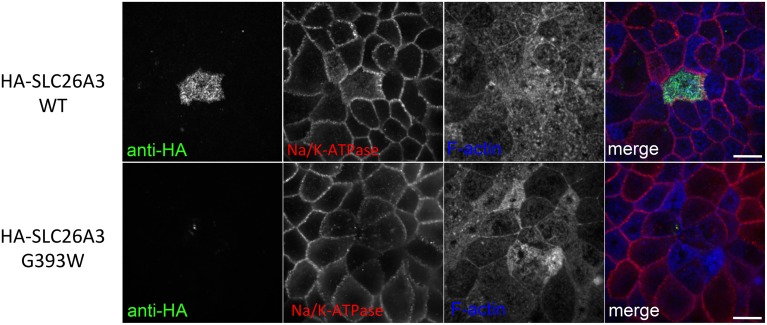
**Localization of wildtype and mutant SLC26A3 polypeptides in confluent, filter-grown Caco-2 cells**. HA-SLC26A3 WT (upper panels) and HA-SLC26A3 G393G (lower panels) in confluent Caco-2 cell monolayers grown on Transwell filters, 72 h post-transient transfection. Cells were stained as indicated with anti-HA, anti-α1-Na,K-ATPase, and phalloidin to detect F-actin. Merged images (with F-actin pseudocolored) shown at right. Scale bar, 10 μm.

However, as shown in Figure 6 (upper panels), 72 h post-transfection of Caco-2 cells previously grown to confluence on filter supports, WT-HA-SLC26A3 is concentrated at or near the apical membrane, without colocalization with basolateral marker Na,K-ATPase. Apical localization was occasional but less reliable in confluent Caco-2 cells grown on glass (not shown) Accumulation of HA-SLC26A3 G393W was barely if at all detectable in similar confluent monolayers of filter-grown Caco-2 cells (Figure 6, lower panels).

### SLC26A3 G393W fails to accumulate in polarized MDCK cells

Since mutant HA-SLC26A3 G393W failed to accumulate in confluent Caco-2 cells on filters, we examined the mutant protein's localization in transiently transfected, confluent, filter-grown MDCK cells.

**Figure 7:**
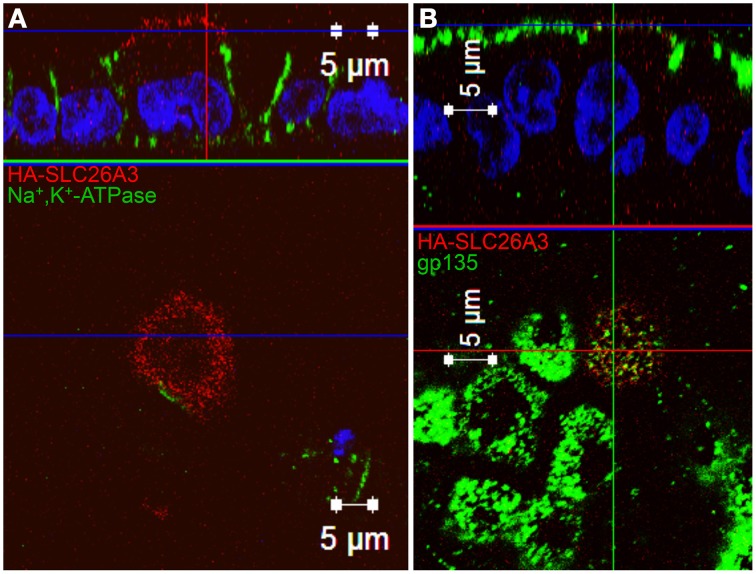
**Localization of wildtype SLC26A3 in filter-grown MDCK cells**. Confluent, filter-grown, MDCK monolayers 48 h after transient transfection with cDNA encoding wildtype HA-SLC26A3. Fixed, permeabilized cells were costained with anti-HA (red) and antibody to the endogenous basolateral marker Na^+^,K^+^-ATPase (**A**, green) or to the endogenous apical marker gp135/podocalyxin (**B**, green). The x-z images at top are sections situated at the horizontal lines in the lower x-y panels. The x-y panels at bottom are sections situated at the blue horizontal lines in the upper x-z panels. Scale bars, 5 μm.

**Figure 8:**
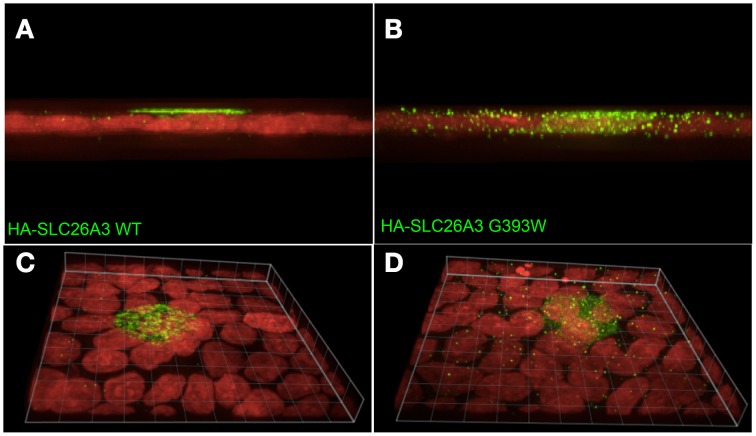
**Localization of wildtype and mutant SLC26A3 polypeptides in confluent, glass-grown MDCK cells**. X-Z confocal sections **(A,C)** and 20°-tilted Z stack of X-Y projections **(B,D)** of confluent glass-grown MDCK monolayers HA-SLC26A3-WT (left panels) and mutant HA-SLC26A3 G393W (Right panels), 72 h post-transient transfection. Anti-HA is shown in green, nuclear stain DRAQ5 in red. Scale bar, 10 μm.

As shown in Figure 7A, wildtype HA-SLC26A3 (red) accumulated in the apical membrane of MDCK cells, a location completely distinct from that of the basolaterally localized Na,K-ATPase (green), but within the same membrane as the endogenous apical marker gp135/podocalyxin (Figure 7B, green). Transfection into polarized MDCK cell monolayers of mutant HA-tagged SLC26A3 G393W cDNA yielded variable results. On occasion, as shown in Figures 8B,D, HA-SLC26A3 G393W did accumulate at low levels throughout the cell, without apparent exclusion from the apical membrane. But cells with any accumulation of the mutant polypeptide were unusual, and contrasted to the exclusively apical localization of HA-SLC26A3 WT polypeptide (Figures 8A,C).

**Figure 9:**
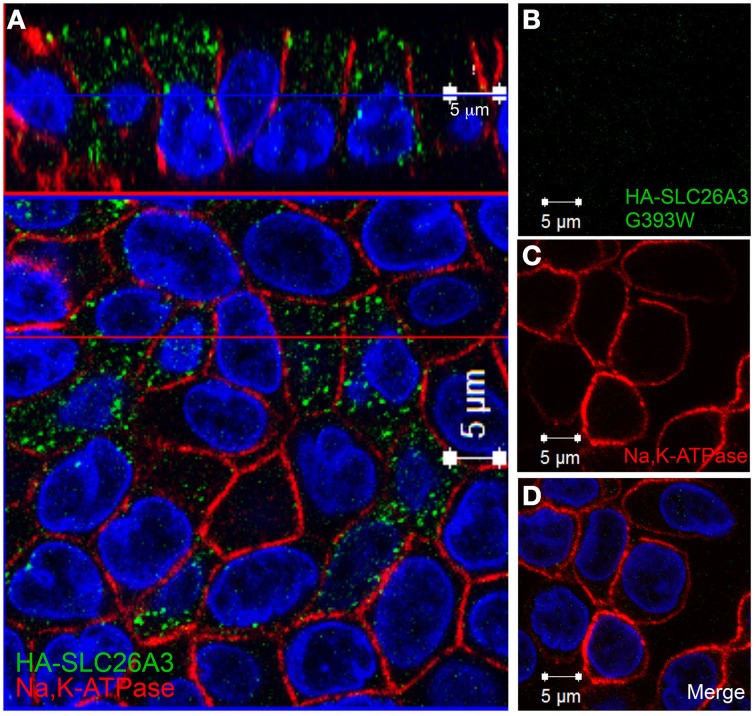
**Localization of wildtype and mutant SLC26A3 in glass-grown MDCK cells**. Confluent, glass-grown MDCK monolayers 48 h after transient transfection with **(A)** cDNA encoding wildtype HA-SLC26A3 or with **(B–D)** cDNA encoding mutant HA-SLC26A3 G393W. Fixed, permeabilized cells were costained with anti-HA (red) and antibody to Na^+^,K^+^-ATPase (red). Nuclei are stained with DAPI. Location of x-z sections with respect to x-y sections is as described for Figure 6. Scale bar 5 μm.

In confluent, semi-polarized MDCK cell monolayers grown on glass, transiently transfected HA-SLC26A3 often was dispersed throughout the cells without preferential localization to either apical or basal peripheral membrane (Figure 9A). However, transiently transfected mutant HA-SLC26A3 G393W did not accumulate above barely detectable levels in any cells observed (Figures 9B–D), likely reflecting diminished mutant protein stability in the setting of the relatively low transfection efficiency characteristic of MDCK cells.

Transient transfection of cDNAs encoding HA-SLC26A3 WT and G393W cDNAs into confluent LLC-PK1 monolayers on glass similarly yielded barely detectable expression of the mutant protein, despite apparent transfection efficiencies of 30–40% as measured by GFP co-transfection (not shown).

## Discussion

We have presented a case of congenital chloride diarrhea (CLD) in a child in whom we have documented the novel SLC26A3 missense mutation G393W. This is the first SLC26A3 mutation reported from Mexico or from Latin America.

SLC26A3 mediates most of human intestinal apical Cl^−^/HCO^−^_3_ exchange responsible for NHE3-coupled NaCl reabsorption (Walker et al., 2008; Alper and Sharma, 2013). Impairment or loss of this function is believed adequate to explain the intestinal and systemic acid-base phenotypes of CLD in neonates. Initial reports of successful treatment of CLD by omeprazole or by sodium butyrate have not proven reproducible in most patients, and lifelong replacement of ongoing fluid and salt losses remains the mainstay of therapy.

SLC26A3 mediates nearly all electroneutral Cl^−^/HCO^−^_3_ exchange in mouse cecum (Alper et al., 2011), and a large part of cecal sulfate/Cl^−^ exchange (Whittamore et al., 2013). In mouse duodenum, SLC26A3 contributes substantially to basal Cl^−^/HCO^−^_3_ exchange activity, and to acid-stimulated HCO^−^_3_ secretion (Singh et al., 2013). In adult humans, deficiency of acid-stimulated duodenal HCO^−^_3_ secretion has been linked to duodenal ulcer, but duodenal ulcer has not been described even as a late-onset feature of congenital chloride diarrhea. Mouse SLC26A3 does not mediate duodenal HCO^−^_3_ secretion stimulated by forskolin (Singh et al., 2013) or PGE2 (Tuo et al., 2006).

SLC26A3 expression leads to modest oxalate uptake into Xenopus oocytes (Chernova et al., 2003), and its genetic deficiency in mouse was recently shown to reduce luminal uptake of oxalate in ileum, cecum, and distal colon of mouse (Freel et al., 2013). However, other evidence strongly suggests that murine intestinal oxalate absorption is mediated entirely through paracellular pathways (Knauf et al., 2011). Interestingly, the chronic renal insufficiency of congenital chloride diarrhea is characterized by nephrocalcinosis (von Kossa-positive, and thus likely calcium phosphate) without nephrolithiasis, in the absence of serum oxalate abnormalities, hypercalciuria or hyperoxaluria. The reappearance of nephrocalcinosis in the transplanted kidney of a CLD patient, however, suggests that the renal calcification reflected chronic systemic volume depletion and alkalosis rather than an intrinsic renal defect (Wedenoja et al., 2008).

Our current results showed that in Xenopus oocytes, SLC26A3 G393W is trafficked to and accumulates at the plasma membrane, where it functions as a mild hypomorphic mutant. However, in HEK-293 cells SLC26A3 G393W is retained intracellularly in a pattern suggestive of endoplasmic reticulum localization, and is greatly reduced in total abundance, without evident surface expression. The mutant phenotype of very low protein accumulation was reproducible among several additional epithelial cell lines. At 24 h post-transfection into subconfluent, not-yet polarized Caco-2 colon carcinoma cells, HA-SLC26A3 WT is localized largely at the cell periphery and less so within the cells, whereas HA-SLC26A3 G393W is largely retained inside the cell. However, at 72 h post-transfection into confluent filter-grown Caco-2 cells, HA-SLC26A3 WT is localized apically, but HA-SLC26A3 G393W is undetectable or present at levels near the threshold of fluorescent detection.

In transiently transfected, polarized, confluent MDCK cells, SLC26A3 polypeptide localizes preferentially to the apical membrane in filter-grown cell monolayers. However, SLC26A3 polypeptide in glass-grown MDCK monolayers is distributed in a pan-cellular punctate pattern suggestive of post-Golgi structures, but with little or no surface expression at either apical or basolateral membrane. Transient transfection of mutant SLC26A3 G393W leads to severely reduced polypeptide abundance in glass-grown MDCK cells, as judged by HA-tag immunofluorescence.

Thus, trafficking and accumulation of both wildtype and G393W mutant SLC26A3 exhibit substantial cell-type specificity and growth condition specificity. In particular, whereas the Xenopus oocyte traffics HA-SLC26A3 mutant G393W to the cell surface, multiple mammalian cell types fail reliably to accumulate the protein and to concentrate it at the plasma membrane. Apical localization of even WT polypeptide is enhanced by growth on permeable filter supports. The mechanisms underlying these cell type and matrix-dependent differences in SLC26A3 mutant trafficking are not clear. However, differences between heterologous membrane transporter trafficking in oocytes and polarized mammalian cells have been noted previously for many proteins (Moeller and Fenton, 2010), including AQP2, CFTR, and AE1/SLC4A1 (Jarolim et al., 1998; Chu et al., 2013).

Little is known of the mechanism for biosynthetic delivery and physiological retrieval and degradation of SLC26A3. SLC26 polypeptides have as yet been associated with no identified β-subunits. Previous heterologous expression tests of subcellular trafficking have also exhibited cell-type specificity. In polarized MDCK cells, apical membrane surface expression of wildtype SLC26A3 required N-glycosylation at least two of the four consensus Asn residues, but surface expression in nonpolarized CHO cells was independent of N-glycosylation (Hayashi and Yamashita, 2012). The C-terminal PDZ recognition motif of SLC26A3 was dispensable for surface expression and basal function in Xenopus oocytes (Chernova et al., 2003) and for acute inhibition by acidic pH_i_ (Wedenoja et al., 2008; Hayashi et al., 2009), but essential for SLC26A3 stimulation signals that elevate intracellular [Ca^2+^] or activate phosphatidylinositol-3-kinase (Lamprecht et al., 2009; Lissner et al., 2010). This PDZ recognition motif of SLC26A3 bound to the third PDZ domain of the cytoskeletal scaffold protein NHERF4 (Lee et al., 2012), an interaction that reduced SLC26A3 surface expression. The PDZ motif also recognized the additional PDZ domain scaffold proteins PDZK1, E3KARP/ NHERF2, and IKEPP in the context of apical lipid rafts, pharmacological disruption of which decreased transporter abundance at the cell surface (Lissner et al., 2010). The role of ubiquitination, and the relative roles of proteasomal and lysosomal pathways in the regulated degradation of SLC26A3 remain unknown.

In the absence of a crystal structure for an SLC26 transmembrane domain, the location of G393 within the polypeptide transmembrane domain is not known. However, G393W is predicted to reside at the exofacial (carboxy-terminal) end of putative transmembrane helix 9 based on hydropathy prediction algorithms. The 45 CLD-associated missense, frameshift, or nonsense mutations reported to date include 28 in the transmembrane domain. The nearest to G393W in the primary structure include G379A, V381Del, and S398F. Nearby (nominally asymptomatic) cSNPs include S398T and S405T. Disease mutations somewhat farther away in the linear sequence include C343Y and D468V. Only 18% of coding mutations in SLC26A3 have been found in the C-terminal cytoplasmic section that includes the STAS domain.

Intestinal SLC26A3 mRNA has been reported to be downregulated in ulcerative colitis and in the IL10 transgenic colitis model (Yang et al., 1998; Lohi et al., 2002), but SLC26A3 protein levels in intestinal tissues of patients with ulcerative colitis were unchanged (Lohi et al., 2002). Polymorphisms in the *SLC26A3* gene have been linked to ulcerative colitis in Japanese and Korean patients (Asano et al., 2009; Yang et al., 2011), but not to Crohn's disease. Polymorphic variants of *SLC26A3* have also been linked to prognosis of patients with colon carcinoma (Dalerba et al., 2011) and to neoadjuvant therapy resistance in HER2-negative breast carcinoma (De Ronde et al., 2013).

What selective pressure might favor retention of *SLC26A3* loss-of-function alleles? SLC26A3 has been shown to interact with the CF transmembrane regulator CFTR. *SLC26A3* hypomorphs with decreased intestinal chloride absorption might in theory exhibit attenuated CF phenotypes in the presence of at least some CFTR mutations, perhaps those of moderate severity. However, a GWAS study of 3763 CF patients failed to reveal *SLC26A3* as a strong modifier gene for the neonatal intestinal CF phenotype of meconium ileus. In contrast, the *NHE3* gene (encoding the NHE3 Na^+^/H^+^ exchanger functionally coupled to SLC26A3) and the evolutionarily related *SLC26A9* gene (highly expressed in apical membrane of mouse stomach and duodenum, Liu et al., 2015) were detected as CF meconium ileus modifier genes (Sun et al., 2012).

The understudied native populations of Latin America may offer new opportunity for discovery and investigation of novel mutations in the *SLC26A3* gene, and in other genetic regulators and modifiers of intestinal salt reabsorption and secretion. Elucidation of the physiological impact of these mutations on the trafficking and function of SLC26A3 and its biochemical and functional polypeptide partners in ion transport should offer new paths to therapeutic intervention in the treatment of diarrheal disease, and insights into trans-epithelial ion transport in other organs.

## Author contributions

SVC and SA conceived and designed the work. FR, SB, BS, ZZ, BW, DD, DS, and JT conducted experiments and acquired data. FR, SB, ZZ, IS, BW, SVC, DD, DS, JT, and SA analyzed and interpreted the data. FR and SA drafted the manuscript. All authors reviewed, critiqued, and helped revise the manuscript, and approved the its final submitted form. All authors agree to be accountable for all aspects of the work in ensuring that questions related to the accuracy or integrity of any part of the work are appropriately investigated and resolved.

## Funding

FR was supported by a fellowship of the Robert Bosch Foundation. Additional support was from NIH DK34854 (Harvard Digestive Diseases Center to WIL and SLA), DK048106 and DK084424 (to WIL), the Ministry of Science and Technology of China grants 2010CB529601 and 2013CB945404 (to BLW), and the Hospital Infantil de Mexico and the Instituto Nacional de Salud (to SVC).

### Conflict of interest statement

The authors declare that the research was conducted in the absence of any commercial or financial relationships that could be construed as a potential conflict of interest.
